# Perissodactyl diversities and responses to climate changes as reflected by dental homogeneity during the Cenozoic in Asia

**DOI:** 10.1002/ece3.6363

**Published:** 2020-06-10

**Authors:** Bin Bai, Jin Meng, Christine M. Janis, Zhao‐Qun Zhang, Yuan‐Qing Wang

**Affiliations:** ^1^ Key Laboratory of Vertebrate Evolution and Human Origins of Chinese Academy of Sciences Institute of Vertebrate Paleontology and Paleoanthropology Chinese Academy of Sciences Beijing China; ^2^ CAS Center for Excellence in Life and Paleoenvironment Beijing China; ^3^ Division of Paleontology American Museum of Natural History New York NY USA; ^4^ Earth and Environmental Sciences Graduate Center City University of New York New York NY USA; ^5^ School of Earth Sciences University of Bristol Bristol UK; ^6^ Department of Ecology and Evolutionary Biology Brown University Providence RI USA; ^7^ College of Earth and Planetary Sciences University of Chinese Academy of Sciences Beijing China

**Keywords:** Asian perissodactyl diversity, Cenozoic, premolar molarization, Ulan Gochu Decline

## Abstract

Cenozoic mammal evolution and faunal turnover are considered to have been influenced and triggered by global climate change. Teeth of large terrestrial ungulates are reliable proxies to trace long‐term climatic changes due to their morphological and physicochemical properties; however, the role of premolar molarization in ungulate evolution and related climatic change has rarely been investigated. Recently, three patterns of premolar molarization among perissodactyls have been recognized: endoprotocrista‐derived hypocone (type I); paraconule–protocone separation (type II); and metaconule‐derived pseudohypocone (type III). These three patterns of premolar molarization play an important role in perissodactyl diversity coupled with global climate change during the Cenozoic in Asia. Those groups with a relatively higher degree of premolar molarization, initiated by the formation of the hypocone, survived into Neogene, whereas those with a lesser degree of molarization, initiated by the deformation of existing ridges and cusps, went extinct by the end of the Oligocene. In addition, the hypothesis of the “Ulan Gochu Decline” is proposed here to designate the most conspicuous decrease of perissodactyl diversity that occurred in the latest middle Eocene rather than at the Eocene–Oligocene transition in Asia, as conventionally thought; this event was likely comparable to the contemporaneous post‐Uintan decline of the North American land fauna.

## Graphical abstract

Three patterns of premolar molarization play an important role in perissodactyl diversity coupled with global climate change during the Cenozoic in Asia. In addition, the hypothesis of the “Ulan Gochu Decline” is proposed to designate the most conspicuous decrease of perissodactyl diversity that occurred in the latest middle Eocene rather than at the Eocene–Oligocene transition in Asia, as conventionally thought.

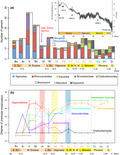

## 1 INTRODUCTION

Living perissodactyls (odd‐toed ungulates) represent the remnants of a major evolutionary sequence and comprise only six genera and 17 species with many in danger of extinction (Nowak & Walker, 1999). However, perissodactyls had a rich, diverse fossil record spanning 56 Myr, and have not only long been used as a strong evidence for evolution since Huxley (e.g., horses), but also for inferring climatic and environmental changes, and their coevolution with such changes (Franzen, 2010; MacFadden, 1992; Mihlbachler, Rivals, Solounias, & Semprebon, 2011; Secord et al., 2012; Simpson, 1951). The most conspicuous fauna turnover during the Paleogene in Asia is considered to have occurred during the Eocene–Oligocene transition, and the Eocene perissodactyl‐dominant faunas were abruptly replaced by the Oligocene rodent/lagomorph‐dominant faunas (Meng & McKenna, 1998). This major faunal turnover, known as “Mongolian Remodelling,” is attributed to climatic changes from the warm, humid Eocene to the cooler and more arid Oligocene (Meng & McKenna, 1998; Zachos, Pagani, Sloan, Thomas, & Billups, 2001). However, there is controversy as to whether this faunal turnover either predates (Wasiljeff, Kaakinen, Salminen, & Zhang, 2020), coincides with (Sun et al., 2014; Zhang, Kravchinsky, & Yue, 2012), or postdates (Kraatz & Geisler, 2010) the Eocene–Oligocene boundary.

Perissodactyls have been considered to have originated from within the radiation of phenacodont condylarths (Radinsky, 1966; Thewissen & Domning, 1992) or to be a sister group to *Radinskya* from the middle Paleocene of China (Holbrook, 2014; McKenna, Chow, Ting, & Luo, 1989), while more recent work has shown cambaytheres from the early Eocene of Indian subcontinent to be more closely related to perissodactyls than either of these previously considered taxa (Rose et al., 2014). Recent research on ancient proteins suggests that crown perissodactyls are the sister group to some extinct South American ungulates among more recent mammals (Welker et al., 2015). Teeth, composed of the hardest vertebrate tissues, are the best preserved material in perissodactyls as in other mammal fossils, and the most sensitive proxy to environmental changes (Mihlbachler et al., 2011; Secord et al., 2012). Previous studies on the dentition of extinct perissodactyls (and other ungulates) have focused on molar crown height, enamel stable isotopes, micro‐mesowear, and overall morphology through the Cenozoic (Ackermans, 2020; Evans & Pineda‐Munoz, 2018; Jernvall, Hunter, & Fortelius, 1996; Mihlbachler et al., 2011; Secord et al., 2012), but little attention has been paid to the evolutionary patterns of premolar molarization in perissodactyls (Butler, 1952; Holbrook, 2015).

Molarization of the premolars generally results in dental homogeneity and increases the grinding area of the dentition; this is especially important for hindgut fermenting ungulates such as perissodactyls as they are highly reliant on oral processing of the food before its initial ingestion (Clauss, Nunn, Fritz, & Hummel, 2009; Fletcher, Janis, & Rayfield, 2010). In contrast, the foregut fermenting artiodactyls, ruminants and camelids, are less reliant on oral processing: they only have partially molarized premolars, and their premolar complexity decreases from P4 to P2 (P1 is usually lost). However, it is unclear what the role of premolar molarization is in perissodactyl evolution and diversity, how perissodactyl diversity and premolar molariform changed through the Cenozoic in Asia, and whether perissodactyl diversity tracked the Cenozoic climatic changes. Here, we show that three recently proposed patterns of premolar molarization in perissodactyls may have played an important role in their response to climatic and environmental changes during the Cenozoic. Further, based on analysis of perissodactyl diversity with an updated Cenozoic timescale of China in Asia, we note that the most distinct change occurred during the latest middle Eocene, rather than at Eocene–Oligocene transition as conventionally considered, and is likely comparable to the contemporaneous post‐Uintan decline of North American land fauna (Prothero, 1994).

## 2 METHODS

### 2.1 Premolar molarization

**FIGURE 1:**
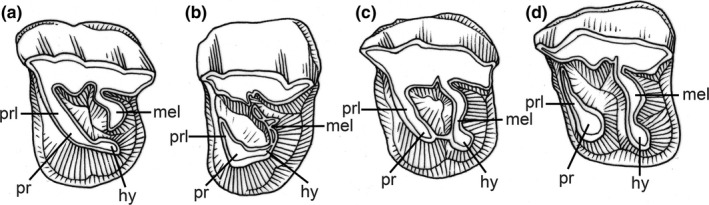
The degree of premolar molarization in perissodactyls as shown by rhinoceros premolars (modified from Qiu and Wang (2007)). (a) Premolariform (assigned value 2); (b) submolariform (assigned value 3); (c) semimolariform (assigned value 4); (d) molariform (assigned value 5). hy, hypocone; mel, metaloph; pr, protocone; prl: protoloph

Five categories of premolar molarization, initially used for rhinoceroses, were assigned to the P2‐4 of perissodactyls: nonmolariform, premolariform, submolariform, semimolariform, and molariform (Qiu & Wang, 2007) (Figure 1). In the premolariform morphology, the hypocone is united with the protoloph, but the metaloph is not completely formed (i.e., is separate from the hypocone) (Figure 1a). In the submolariform morphology, the metaloph is completely formed (connected to the hypocone) and united with the protoloph on the lingual side (Figure 1b). In the semimolariform morphology, the protocone and hypocone are distinctly separate, but still connected by an enamel ridge (Figure 1c). In the molariform morphology, the protoloph and metaloph are completely separate (Figure 1d). Numbers from one to five were assigned to the five categories, respectively, similar to those proposed by Prothero (2005). These numbers were assigned to each premolar (P2‐4) according to their degree of premolar molarization (Appendix Tables A1, A2, A3). The mean values for P2‐4 were then calculated, which represent the degree of premolar molarization of each genus. We then calculated the mean value of the degree of premolar molarization in each perissodactyl family during each Asian Land Mammal Age (ALMA) (Appendix Table A2).

Among perissodactyls, brontotheres are characterized by bunodont–lophodont teeth with relatively weak transverse lophs on the upper premolars, unlike the condition in other perissodactyls that are more strictly lophodont, and so we characterized their teeth in a somewhat different fashion. We assigned premolariform (value 2) to the brontotheres with a “lingual crest” (Mihlbachler, 2008) on the upper premolars, semimolariform (value 4) to those with distinct hypocones on the “lingual crest,” and molariform (value 5) to those with distinct hypocones completely separated from the protocone.

Three patterns of premolar molarization among perissodactyls have been recognized, including an endoprotocrista‐derived hypocone (type I), paraconule–protocone separation (type II), and a metaconule‐derived pseudohypocone (type III) (Bai, Meng, Mao, Zhang, & Wang, 2019; Holbrook, 2015). The premolar molarization in most perissodactyls was initiated by the type I pattern, which means that the hypocone developed from a crista posterior to the protocone (endoprotocrista) (Holbrook, 2015). However, the P2‐4 of deperetellid tapiroids, the P3 of the Eocene equids, and the P2 of the hyrachyid *Metahyrachyus* adopted the type II pattern, which entails the separation of the paraconule from the protocone, and the paraconule is enlarged and lingually extended (Bai et al., 2019). Only amynodontid rhinocerotoids had the type III pattern, where the metaconule is separated from the protocone and is lingually extended (Bai et al., 2019). In contrast, the molar hypocones evolved either from the postprotocingulum, as in most eutherians, or from the metaconule, as in artiodactyls (Hunter & Jernvall, 1995).

### 2.2 Hypsodonty

Different methods for calculating the Hypsodonty Index (HI) have been proposed (Fortelius et al., 2002; Janis, Damuth, & Theodor, 2002; MacFadden, 1992; Van Valen, 1960), but all entail the comparison of the unworn tooth crown height with some other dental linear measurement. Here, we follow Fortelius et al. (2002) in using HI as a ratio of height to length of the second molar (upper or low), although the deficiency of using length instead of width has been noticed (Damuth & Janis, 2011). Three classes of hypsodonty were proposed by Fortelius et al. (2002) based on the following criteria: brachydont teeth with a ratio of less than 0.8 (assigned a value of 1); mesodont teeth with a ratio of 0.8–1.2 (assigned a value of 2); and hypsodont teeth with a ratio of greater than 1.2 (assigned a value of 3). For the Neogene perissodactyls from Asia, the classes of hypsodonty for almost all genera can be found in the NOW (New and Old World) database (NOW: http://www.helsinki.fi/science/now/) (Appendix Tables A1 and A4). However, data for the classes of hypsodonty of Paleogene perissodactyls in Asia are almost entirely lacking. We considered all Eocene perissodactyls to have brachydont teeth except for the amynodontids *Huananodon* (mesodont) (You, 1977) and *Hypsamynodon* (hypsodont) (Gromova, 1954).

### 2.3 Taxa selection

The Paleogene Asian faunas and comparisons were mainly based on references from the literature (Li & Ting, 1983; Russell & Zhai, 1987; Tong, Zheng, & Qiu, 1995; Wang et al., 2019). The Neogene Asian faunas were mainly based on Savage and Russell (1983) with updated data from Deng, Hou, and Wang (2019a) and Qiu et al. (2013). Indeterminate taxon identifications and taxonomic modifications such as “cf.” or “?” or some cases of “sp.” were ignored in our analyses. If more than one species of a genus was known from an ALMA, the type species of the genus was selected; otherwise, the most common and well‐preserved species was selected if the type species of the genus was absent during the period in Asia. If intraspecific variation was present, the characteristics of the dentition of the holotype were followed. In the middle Miocene, four genera of elasmotheres were considered to be synonyms of *Hispanotherium* (Deng & Chen, 2016); however, we treated them all as valid genera pending the discovery of more complete material. The subgenera of *Hipparion* from the late Neogene have been elevated to generic levels in recent analyses (Bernor, Wang, Liu, Chen, & Sun, 2018; Sun, Zhang, Liu, & Bernor, 2018a), and we followed this taxonomy.

## 3 RESULTS AND DISCUSSION

**FIGURE 2:**
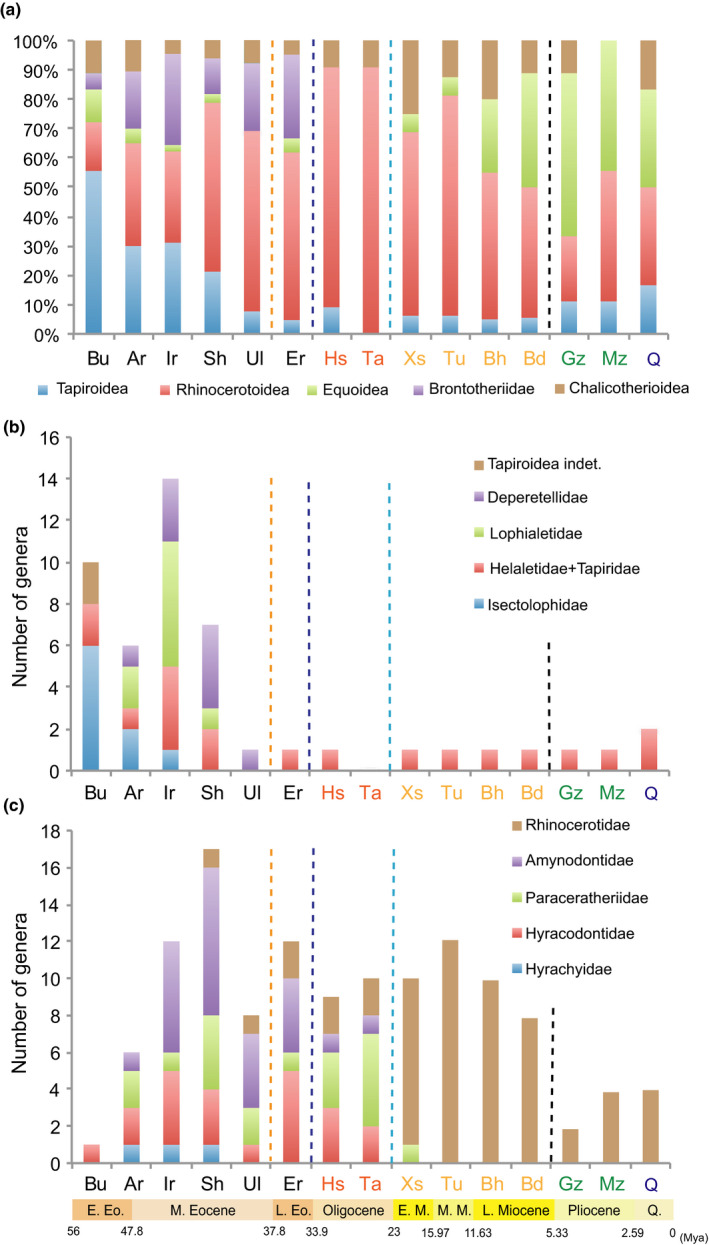
Asian perissodactyl composition (in percentage of genera) and diversity of Tapiroidea and Rhinocerotoidea during the Cenozoic. (a) Histogram showing the composition of five main groups of perissodactyls during the Cenozoic in Asia. (b) Histogram showing the diversity and composition of Asian Tapiroidea during the Cenozoic. (c) Histogram showing the diversity and composition of Asian Rhinocerotoidea during the Cenozoic. Bu, Bumbanian; Ar, Arshantan; Ir, Irdinmanhan; Sh, Sharamurunian; Ul, Ulangochuian; Er, Ergilian; Hs, Hsandagolian; Ta, Tabenbulukian; Xs, Xiejian and Shanwangian; Tu, Tunggurian; Bh, Bahean, Bd, Baodean; Gz, Gaozhuangian; Mz, Mazegouan; Q, Quaternary

**FIGURE 3:**
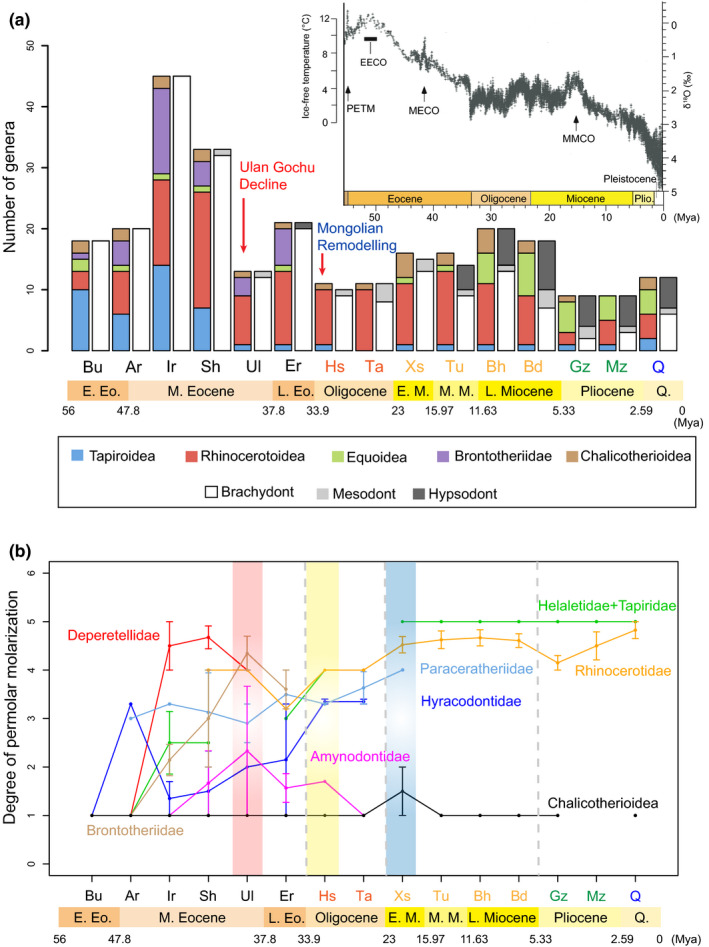
Cenozoic Perissodactyl diversity in Asia and the degree of premolar molarization in different perissodactyl lineages. (a) Perissodactyl diversity and dental hypsodonty in relation to global climatic change (modified from Zachos et al. (2008)) with the most conspicuous decrease of diversity occurring during the latest middle Eocene (“Ulan Gochu Decline”); (b) degree of premolar molarization as represented by the mean value and standard error in different perissodactyl lineages during the Cenozoic in Asia. Equoids are excluded because they were scarce in Asia during the Paleogene. The red, yellow, and blue bars show the time periods of the “Ulan Gochu Decline,” “Mongolian Remodelling,” and the beginning of the Neogene, respectively. The degree of premolar molarization is assigned into five categories (Qiu & Wang, 2007): nonmolariform (1), premolariform (2), submolariform (3), semimolarifom (4), and molariform (5). EECO, Early Eocene Climatic Optimum; MECO, Mid‐Eocene Climatic Optimum; MMCO, Mid‐Miocene Climatic Optimum; and PETM, Paleocene–Eocene Thermal Maximum. The abbreviations along the horizontal axis are Asian Land Mammal Ages as in Figure 2

Taking advantages of the updated Cenozoic timescale in China (Deng et al., 2019a; Wang et al., 2019), and revisions of perissodactyl fossils from China (Bai, Wang, Li, et al., 2018; Deng & Chen, 2016), we compiled a count of the genera of the Cenozoic perissodactyls from Asia and calculated their premolar molarization values (Figures 2 and 3) (Appendix Tables A1 and A3). In general, perissodactyl generic diversity fluctuated in relation to paleoclimatic changes (Bai, Wang, Li, et al., 2018; Zachos, Dickens, & Zeebe, 2008). At the beginning of the early Eocene, perissodactyls had a relatively high diversity, which is consistent with the notion that different lineages of perissodactyls diverged as early as the earliest Eocene during Paleocene–Eocene Thermal Maximum (PETM) (Bai, Wang, & Meng, 2018b). An abrupt increase of diversity from the Arshantan to the Irdinmanhan is likely related to the rising temperatures of the Mid‐Eocene Climatic Optimum (MECO), but the high diversity in the Irdinmanhan may be biased by the overestimation of generic numbers (Bai, Wang, Li, et al., 2018). Deperetellids, helaletids, and paraceratheriids attained a relatively high or the maximum degree of premolar molarization (mean values ranging from 2.5 to 4.7) in the middle Eocene ALMA, the Irdinmanhan and Sharamurunian (Figure 3b) (Appendix Table A4); this may reflect an increasing ability to process larger amounts of low quality vegetation and the adoption of a relatively more open habitat (Bai, Wang, Li, et al., 2018; Bai, Wang, & Meng, 2018c; Gong et al., 2019, 2020), following the temperature decline after the MECO and the intense of seasonality (Figueirido, Janis, Perez‐Claros, De Renzi, & Palmqvist, 2012; Janis, 1989). However, the degree of premolar molarization shows a degree of fluctuation rather than an average sustained increase at the family level during the Paleogene. It is noteworthy that a few amynodontids possessed mesodont or hypsodont teeth in the middle‐late Eocene, when artiodactyls with teeth that were more lophodont and higher‐crowned (e.g., the oreodont *Leptauchenia* and the hypertragulid *Hypisodus*) also appeared in North America (Janis, 2000). After the Irdinmanhan, the tapiroid‐dominant perissodactyl faunas were gradually replaced by the rhinocerotoid‐dominant ones (Figure 2a–c), and the diversity of perissodactyls generally decreased. However, the most conspicuous event occurred between the Sharamurunian and the Ulangochuian (~39.9 Mya), rather than at the Eocene–Oligocene transition (EOT) (33.9 Mya), when the generic diversity of perissodactyls was reduced from around 33 to 13 (Figure 3a): Lophialetid tapiroids became extinct, deperetellids were reduced from four genera to a single genus (Figure 2b), and rhinocerotoids also suffered (Figure 2c). Similarly, the entire mammalian fauna from China showed a similar abrupt decrease after the Sharamurunian in terms of number of both species and genera (Wang, Meng, Ni, & Li, 2007). We named this event the “Ulan Gochu Decline,” which is likely comparable to the contemporaneous post‐Uintan decline of the North American land fauna (Berggren & Prothero, 1992; Prothero, 1994; Stucky, 1990) and the beginning of the White River Chronofauna in the late Duchesnean (Woodburne, 2004). The mammalian fauna turnover in the late Duchesnean was considered to have had more influence on the North American fauna than did events at the EOT (Berggren & Prothero, 1992; Meng & McKenna, 1998). The “Ulan Gochu Decline” was probably related to the sustained cooling following the MECO. The diversity of perissodactyls somewhat increased in the late Eocene (Ergilian) when the temperature rose again slightly (Figure 3a).

The Asian mammalian fauna turnover during the EOT is known as the “Mongolian Remodelling,” with the perissodactyl‐dominant fauna replaced by the rodent/lagomorph‐dominant fauna, a turnover that was attributed to the dramatic drop of temperature at the end of the Eocene (Meng & McKenna, 1998). Recent integrated analyses, however, suggest that the faunal turnover predated the Eocene–Oligocene boundary (Wasiljeff et al., 2020). The climatic deterioration during the EOT also affected primates in southern Asia, favoring the survival of strepsirhines over haplorhines (Ni, Li, Li, & Beard, 2016). In North America, primates (all nonanthropoids) were in decline through the middle Eocene and were essentially extinct by the late Eocene, although a single taxon is known from the latest Oligocene/earliest Miocene (Gunnell, Rose, & Rasmussen, 2008). Among perissodactyls, only brontotheres went extinct during the transition in Asia, as they did in North America (Figures 2a and 3a). The mean value of premolar molarization in hyracodontids gradually increased from 1.4 in the middle Eocene to 3.4 in the late Oligocene, approaching a moderate extent before their extinction by the end of Oligocene (Figure 3b) (Appendix Table A2). Amynodontids reached a peak of premolar molarization in the late middle Eocene with a relatively low value (mean value 2.3), which then decreased gradually to the lowest value (value 1) by the end of the Oligocene before their extinction (Figure 3b). In contrast, paraceratheriids, rhinocerotids, and tapirids, which had higher values of premolar molarization (>3), all survived into the Neogene (Figure 3b), with the implication that this greater degree of premolar molarization contributed to their advantage over those with lower values during the Oligocene/Miocene transition. In addition, premolar molarization type I was likely more advantageous than types II and III, as inferred from the fact that the groups with latter two types of dentitions went extinct before the end of the Paleogene, while almost all perissodactyls with type I dentitions survived into Neogene. Furthermore, the cascade of premolar molarization in a species varied in different groups, such as the premolars of paraceratheres becoming molarized from anterior to posterior teeth (Qiu & Wang, 2007), whereas those of tapiroids took place from posterior to anterior teeth.

In short, the Eocene mammal faunas from Asia showed two pulses of decline in diversity that may be related to global climatic changes. The first one (the “Ulan Gochu Decline”), comparable to the post‐Uintan decline of North American land fauna, took place after the MECO when temperatures declined slowly, and was reflected most clearly in changes in the diversity of perissodactyls. The second one (the “Mongolian Remodelling”), comparable to the European “Grande Coupure,” was at the EOT and may have been a response to the sudden global drop of temperature. It is noteworthy that the endemic Asian taxa sporadically dispersed to North America during the Paleogene, but there was apparently little dispersal in the opposite direction (Beard, 1998). A very few taxa of Asian perissodactyls, such as early equids and palaeotheres, are considered to have dispersed from North America or Europe to Asia during the Paleogene (Bai, 2017; Bai et al., 2018b; Woodburne, 2004), so the immigrants would have had limited impact on the Paleogene Asian perissodactyl diversity.

In the early Miocene, rhinocerotids replaced paraceratheriids and dominated the perissodactyl groups (Figure 2c). The Chinese rhinocerotid diversity and responses to the Neogene climatic change have been investigated by Deng and Chen (2016) and Deng and Downs (2002), and it is not necessary to replicate them here. However, the following statements need to be addressed: The appearance of the rhinocerotid *Hispanotherium* with hypsodont teeth in the middle Miocene indicates a more abrasive diet and a slightly more open and drier habitat, which is enhanced in the late Miocene with the spread of Old World savanna palaeobiome (Kaya et al., 2018). The decrease of rhinocerotid diversity and the rise of equid diversity during the mid‐late Miocene transition have been mainly attributed to the cooling event and the dispersal of hipparionine equids from North America to Eurasia in the late Miocene (MacFadden, 1992). During the late Miocene, the hypsodont equids and rhinocerotids coexisted with brachydont or mesodont ones (Figure 3a; Appendix Table A1), but the proportion of hypsodont groups gradually increased. The decreased diversity of rhinocerotids in the early Pliocene was probably due to the expansion of C_4_ grasses (Han, Wang, & Liu, 2002), although the climate was relatively warm and humid except in the high altitude, cold Tibetan Plateau (Deng et al., 2019b). The hypsodont equids became the dominant taxa among perissodactyl groups in the Pliocene (Figure 3a; Appendix Table A1). In addition, the distribution of Chinese mammals has also been influenced by the East Asian Monsoon (Qiu & Li, 2005), which was probably initiated in the Eocene and intensified in the late Miocene, driven by the uplift of the Tibetan Plateau (Qiu & Li, 2005; Quan et al., 2014). The degree of premolar molarization in tapirids, rhinocerotids, and equids remained high (mean value >4.2) and virtually stable through the Neogene, and the main modification of the teeth in the latter two clades lay in increasing the height of the molar/premolar crowns and the complexity of the occlusal enamel (Figure 3, Appendix Table A4) (Deng & Chen, 2016; Famoso, Davis, Feranec, Hopkins, & Price, 2016; Fortelius et al., 2002; Simpson, 1951). Paraceratheriids, with a relatively lesser degree of premolar molarization and persistently mesodont teeth, disappeared after the early Miocene, possibly related to the competition from proboscideans and their effects on the environment (Prothero, 2013).

Among perissodactyls, chalicotheres were conservative with almost unmolarized premolars, and they had a relatively low diversity from the early Eocene to the early Pleistocene (Figure 3). Their extinction in the Plio‐Pleistocene may be attributed to climatic change.

**FIGURE 4:**
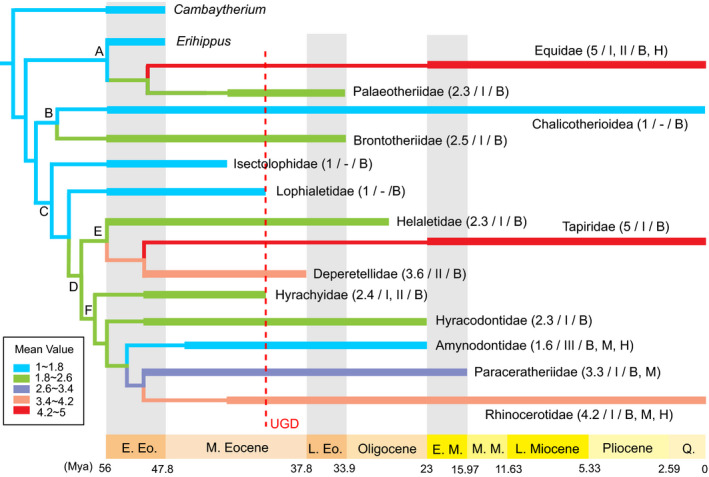
Phylogeny and distribution of perissodactyls from Asia showing the ancestral mean values of degrees of premolar molarization. A proposed phylogeny of Perissodactyla was combined from McKenna and Bell (1997), Rose et al. (2014), and updated data. Each family name is followed by information (in parentheses) listing (separated by slashes): the mean value of premolar molarization, the pattern of premolar molarization, and the hypsodonty level. The ancestral mean values were reconstructed using the parsimonious criterion with the linear cost assumption in Mesquite 3.6 (Maddison & Maddison, 2018). The letters from A to E at the nodes refer to the following clades: A for Equoidea, B for Selenida, C for Ceratomorpha, D for crown Ceratomorpha, E for Tapiroidea, and F for Rhinocerotoidea. B, brachydont; M, Mesodont; H, Hypsodont; UGD, “Ulan Gochu Decline.”

To investigate the evolution and relationships of variable degrees of premolar molarization among different perissodactyl lineages, mean values of premolar molarization degrees at ancestral nodes were reconstructed on a phylogenetic tree of Perissodactyla by a parsimonious criterion (Figure 4). The mean values at the ancestral nodes generally increased from the basal nodes to more derived nodes. The mean value at the equoid ancestral node (Node A) is low and then increased in later equoids. Chalicotheres and brontotheres diverged from a common ancestor (Node B) with a low mean value. Among Ceratomorpha (Node C), the stem taxa Isectolophidae and Lophialetidae had low mean values, and their premolars remained unmolarized, while the common ancestor of crown Ceratomorpha (Node D) had an increasing degree of premolar molarization. The mean value of premolar molarization increased toward the Tapiridae–Deperetellidae clade from the ancestral node with the Helaletidae (Node E), and Deperetellidae was the first group to evolve a relatively high degree of premolar molarization. Among Rhinocerotoidea (Node F), the mean values of premolar molarization were relatively low in basal Hyrachyidae and Hyracodontidae and gradually increased in the lineage leading to the Paraceratheriidae and Rhinocerotidae. However, Amynodontidae had a decreasing mean value from the ancestral node. The explanation for this reverse is uncertain and would be resolved by better data on basal taxa and a more comprehensive phylogenetic analysis of Perissodactyla in near future.

## 4 CONCLUSIONS

Different patterns of premolar molarization likely played an important role in patterns of perissodactyl diversity in concert with global climatic changes during the Cenozoic in Asia. Most perissodactyls with a relatively higher degree of premolar molarization, and with this molarization formed by the hypocone, survived into Neogene; whereas those with less molarized premolars, and with molarization initiated by the deformation of existing ridges and cusps, went extinct by the end of Oligocene. Although perissodactyl diversity has generally declined since the early middle Eocene, the most conspicuous decrease (the Ulan Gochu Decline) occurred during the latest middle Eocene rather than at the Eocene–Oligocene boundary in Asia, as conventionally thought. However, whether this event also impacted other fossil mammals in Asia needs further investigation.

## CONFLICT OF INTEREST

None declared.

## AUTHOR CONTRIBUTIONS

**Bin Bai:** Data curation (equal); formal analysis (equal); funding acquisition (equal); investigation (equal); methodology (equal); project administration (equal); software (lead); visualization (lead); writing–original draft (lead); writing–review and editing (lead). **Jin Meng:** Methodology (equal); project administration (equal); supervision (equal); writing–original draft (supporting). **Christine M. Janis:** Formal analysis (equal); investigation (equal); methodology (equal); writing–original draft (supporting); writing–review and editing (supporting). **Zhao‐Qun Zhang:** Data curation (equal); formal analysis (equal); funding acquisition (equal); methodology (equal). **Yuan‐Qing Wang:** Funding acquisition (equal); project administration (equal); supervision (equal).

## Data Availability

All relevant data are within the manuscript and Appendix 1.

## APPENDIX 1

**TABLE A1:** Values of the degree of premolar molarization and Hypsodonty in Cenozoic perissodactyls in Asia

	Family	Genus	P2	P3	P4	M.	N.	H.	Ref.
Bumbanian									
Tapir.	'Isecto.'	*Meridiolophus*				?	6		Bai, Wang, Meng, Li, and Jin (2014)
		*Chowliia*	1	1	1	1			Tong and Wang (2006)
		*Homogalax*		1	1	1			Tong and Wang, (2006)
		*Karagalax*		1	1	1			Maas, Hussain, Leinders, and Thewissen (2001)
		*Ampholophus*	1	1	1	1			Tong and Wang (2006), Wang and Tong (1996)
		*Orientolophus*				?			Bai et al. (2018b), Ting (1993)
	Hela.	*Heptodon*				1	2		Xu, Yan, Zhou, Han, and Zhong (1979)
		*Vastanolophus*				?			Smith et al. (2015)
	Loph.	*Minchenoletes*			1	1	2		Wang et al. (2011)
		*Ampholophus*	1	1	1	1			Tong and Wang (2006)
	Indet.	*Cambaylophus*				?	2		Kapur and Bajpai (2015)
		*Orientolophus*				?			Ting (1993)
Rhino.	Hyraco.	*Pataecops*	1	1	1	1	3		Radinsky (1965), Wang et al. (2011)
	Indet.	*Minchenoletes*			1	1			Wang et al. (2011)
		*Yimengia*							
Equ.	Equidae	*Erihippus*				?	2		Bai et al. (2018b), Ting (1993)
		*Ghazijhippus*	1	1	1	1			Missiaen and Gingerich (2014)
Bron.		*Danjiangia*	1	1	1	1	1		Bai et al. (2018b), Wang (1995)
Ch.	Eomo.	*Protomoropus*			1	1	2		Hooker and Dashzeveg (2004)
		*Pappomoropus*				?			Tong and Wang (2006)
Arshantan									
Tapir.	'Isecto.'	*Gandheralophus*		1	1	1	2		Missiaen and Gingerich (2012)
		*Isectolophus*		1	1	1			Lucas, Holbrook, and Emry (2003)
	Helal.	*Heptodon*				1	1		Qi (1987)
	Loph.	*Schlosseria*	1	1	1	1	2		Radinsky (1965)
		*Parabreviodon*	1	1	1	1			Reshetov (1975)
	Dep.	*Irenolophus*	1	1	1	1	1		Bai et al. (2019)
Rhino.	Hy.	*Hyrachyus*				3	1		Qi (1987)
	Hyraco.	*Ephyrachyus*	4	3	3	3.3	2		
		*Triplopus*				?			
	Para.	*Pappaceras*	3	3	3	3	2		Lucas, Schoch, and Manning (1981), Wang, Bai, Meng, and Wang (2016), Wood (1963)
		*gen. nov.*				?			
	Amy.	*Euryodon*				?	1		Xu et al. (1979)
	Indet.	*Yimengia*					1		
Equ.	Equidae	*Propalaeotherium*				?	1		Zdansky (1930)
Bron.		*Desmatotitan*				?	4		Qi (1987)
		*Paleosyops*				1			Russell and Zhai (1987), Xu et al. (1979)
		*Balochititanops*			1	1			Missiaen, Gunnell, and Gingerich (2011)
		*Eotitanops*			1	1			Missiaen et al. (2011)
Ch.	'Eomo.’	*Litolophus*	1	1	1	1	2		Bai, Wang, and Meng (2010), Colbert (1934), Missiaen and Gingerich (2012), Radinsky (1964)
		*Grangeria*	1	1	1	1			Zdansky (1930)
Irdinmanhan									
Tapir.	Isecto.	*Sastrilophus*		1		1	1		Sahni and Khare (1971)
	Helal.	*Paracolodon*	4	4	4	4	4		Bai, Wang, Mao, and Meng (2017), Matthew and Granger (1925c)
		*Desmatotherium*	3	3	3	3			Bai et al. (2017)
		*Helaletes*				2			Gabunia (1961), Radinsky (1965)
		*Rhodopagus*		1	1	1			
	Loph.	*Lophialetes*	1	1	1	1	6		Matthew and Granger (1925c), Radinsky (1965)
		*Simplaletes*				?			Qi (1980)
		*Zhongjianletes*				?			Ye (1983)
		*Schlosseria*	1	1	1	1			Tong and Lei (1984)
		*Kalakotia*	1	2	1	1.3			Ranga Rao (1972)
		*Eoletes*	1	1	1	1			Biryukov (1972)
	Dep.	*Teleolophus*	4	4	4	4	3		Radinsky (1965), Tong and Lei (1984)
		*Pachylophus*				?			Tong and Lei (1984)
		*Deperetella*				5			Chow, Li, and Zhang (1973), Reshetov (1979)
Rhino.	Hy.	*"Hyrachyus"*	4	3	3	3.3	1		Huang and Wang (2002)
	Hyraco.	*Triplopus*	1	1	1	1	4		Matthew and Granger (1925c), Radinsky (1967)
		*Prohyracodon meridionale*	1	2	2	1.7			Chow et al. (1973), Chow and Xu (1961), Xu et al. (1979)
		*Caenolophus* sp.							
		*Ilianodon*				?			Chow and Xu (1961)
	Para.	*Forstercooperia*	4	3	3	3.3	1		Chow et al. (1973), Sahni and Khare (1973), Wang, Bai, Meng, and Wang (2018), Wood (1938)
	Amy.	*Rostriamynodon*	1	1	1	1	6		Wall and Manning (1986)
		*Sianodon*			1	1			Chow and Xu (1965), Xu et al. (1979)
		*Lushiamynodon*			1	1			Chow and Xu (1965)
		*Amynodon*				1			Chow, Xu, and Zhen (1964)
		*Sharamynodon*				1			Zheng (1978)
		*Teilhardia*? sp.				?			Zheng, Tang, Zhai, Ding, and Huang (1978)
	Indet.	*Breviodon*				?	2		Huang (1982), Radinsky (1965)
		*Yimengia*	1	1	1	1			Wang (1988)
Equ.	Equidae	*Gobihippus*				?	1		Dashzeveg (1979)
Bron.		*Microtitan*	1	1	1	1	14		Granger and Gregory (1943), Mihlbachler (2008)
		*Hyotitan*				?			Granger and Gregory (1943), Mihlbachler (2008)
		*Metatelmatherium*	2	2	2	2			Granger and Gregory (1943), Mihlbachler (2008)
		*Protitian*	2	2	2	2			Granger and Gregory (1943), Mihlbachler (2008), Xu et al. (1979)
		*Gnathotitan*	2	2	2	2			Granger and Gregory (1943), Mihlbachler (2008)
		*Metatitan*	4	4	4	4			Granger and Gregory (1943), Mihlbachler (2008)
		*Desmatotitan*				?			Granger and Gregory (1943), Mihlbachler (2008)
		*Acrotitan*				?			Ye (1983)
		*Epimanteoceras*	4	4	2	3.3			Granger and Gregory (1943), Mihlbachler (2008)
		*Arctotitan*	1	1		1			Wang (1978)
		*Nanotitanops*	2	2	2	2			Qi and Beard (1996), Qi and Beard (1998)
		*Mulkrajanops*	2	2		2			Kumar and Sahni (1985), Mihlbachler (2008)
		*Pakotitanops*				?			West (1980)
		*Palaeosyops* sp.							Gabounia (1977)
Ch.	'Eomo'	*Eomoropus*				1	2		Chow et al. (1973)
		*Lunania*				?			Chow (1957)
Sharamurunian									
Tapir.	Hela.	*Colodon*		4	4	4	2		Takai (1939)
		*Rhodopagus*?				1			Matthew and Granger (1925a), Radinsky (1965)
	Loph.	*Simplaletes*				?	1		Zhang and Qi (1981)
	Dep.	*Deperetella*	4	5	5	4.7	4		Matthew and Granger (1925a), Radinsky (1965)
		*Diplolophodon*		5	5	5			Zdansky (1930), Zong, Chen, Huang, and Xu (1996)
		*Teleolophus*				4			Zong et al. (1996)
		*Bahinolophus*	5	5	5	5			Tsubamoto, Egi, Takai, Sein, and Maung (2005)
Rhino.	Hy.	*Akauhyus*			1	1	1		Huang and Qi (1982), Kordikova (1998)
	Hyraco.	*Triplopus*?				?	3		Matthew and Granger (1925a), Radinsky (1965)
		*Prohyracodon major*	2	2	2	2			Zong et al. (1996)
		*Lijiangia*			1	1			Zong et al. (1996)
	Para.	*Juxia*	4	4	2	4.7	4		Qiu and Wang (2007)
		*Imequincisoria*							Wang (1976), Zhai (1977)
		*Forstercooperia*	3	3	2	2.7			Qiu and Wang (2007), Wang et al. (2018), Wang (1976)
		*Urtinotherium*	2	2	2	2			Chow (1958), Qiu and Wang (2007)
	Amy.	*Sharamynodon*	1	1	1	1	8		Osborn (1936)
		*Sianodon*	1	1	1	1			Xu (1965), Xu (1966), Xu (1978)
		*Lushiamynodon*				?			Xu (1966)
		*Gigantamynodon*				?			Xu (1966)
		*Caenolophus*		1	1	1			Matthew and Granger (1925a), Shi (1989)
		*Huananodon hui*			5	5		2	You (1977)
		*Paramynodon*	1	1	1	1			Colbert (1938)
		*Procadurcodon*	1	1		1			Gromova (1960)
	Rh.	*Guxia simplex*	4			4	1		You (1977)
	indet.	*Breviodon*				?	2		Tong and Wang (1980), Zong et al. (1996)
		*Indolophus*	1	2	2	1.7			Pilgrim (1925), Radinsky (1965)
Equ.	Palae.	*Lophiohippus*				?	1		Bai (2017)
Bron.		*Rhinotitan*	4	4	4	4	4		Granger and Gregory (1943), Mihlbachler (2008)
		*Dianotitan*	4	4	4	4			Chow and Hu (1959), Chow, Zhang, and Ding (1974)
		*Sivatitanops*			1	1			Mihlbachler (2008), Pilgrim (1925)
		*Bunobrontops*				?			Holroyd and Ciochon (2000)
Ch.	'Eomo.'	*Eomoropus*			1	1	2		Shi (1989), Tsubamoto et al. (2005), Zdansky (1930)
		*Grangeria*				1			Zdansky (1930)
Ulangochuian									
Tapir.	Dep.	*Teleolophus*		4	4	4			Radinsky (1965)
Rhino.	Hyraco.	*Ulania*		2		2	1		Qi (1990)
	Para.	*Juxia*		3	2	2.5	2		Qi and Zhou (1989)
		*Urtinotherium*	4	3	3	3.3			Qiu and Wang (2007)
	Amy.	*Amynodontopsis*	1	1	1	1	4		Wall (1981)
		*Amynodon*				1			Qi (1975)
		*Paracadurcodon*				?			Xu (1966)
		*Huananodon hypsodonta*	5	5		5		2	You (1977)
	Rh.	*Guxia youjiangensis*	4	4	4	4	1		You (1977)
Bron.		*Pachytitan*	4	4	4	4	3		Granger and Gregory (1943), Mihlbachler (2008)
		*Titanodectes*				?			Granger and Gregory (1943), Mihlbachler (2008)
		*Embolotherium*	4	5	5	4.7			Granger and Gregory (1943), Mihlbachler (2008), Qi (1975)
Ch.	Chal.	*Schizotherium*				1	1		Zhang (1976)
Ergilian									
Tapir.	Hela.	*Paracolodon*	4	4	1	3	1		Bai et al. (2017), Matthew and Granger (1925b)
Rhino.	Hyraco.	*Ardynia*	5	3	3	3.3	5		Bai, Wang, and Zhang (2018d), Matthew and Granger (1923)
		*Proeggysodon*				?			Bai and Wang (2012)
		*Prohyracodon*				?			Dashzeveg (1991)
		*Armania*	1	1	1	1			Dashzeveg (1991)
		*Guangnanodon*							Wang, Bai, Gao, Huang, and Wang (2013)
	Para.	*Urtinotherium*	4		3	3.5	1		Qi (1989), Qiu and Wang (2007), Tang (1978)
	Amy.	*Zaisanamynodon*	1	1	1	1	4		Lucas, Emry, and Bayshashov (1996)
		*Gigantamynodon*	1		3	2			Gromova (1954), Tang (1978), Xu (1961)
		*Cadurcodon*	1	1	3	1.7			Gromova (1954), Xu (1961)
		*Hypsamynodon*				?		3	Gromova (1954)
	Rh.	*Symphysorrachis*				?	2		Beliayeva (1954)
		*Ronzotherium*	4.5	2.5	2.5	3.2			Dashzeveg (1991), Heissig (1969)
Equ.	Palae.	*Qianohippus*	5	1	1	2.3	1		Bai (2017), Miao (1982)
Bron.		*Embolotherium andrewsi*	4	4	4	4	6		Granger and Gregory (1943), Mihlbachler (2008), Qi (1975)
		*Parabrontops*	2	5	5	4			Granger and Gregory (1943), Mihlbachler (2008), Qi (1975)
		*Pygmaetitan*	2	2	2	2			Miao (1982)
		*Metatitan*	4	4	4	4			Yanovskaya (1980)
		*Protembolotherium*	4	4	4	4			Yanovskaya (1954)
		*Titanodectes*				?			Granger and Gregory (1943)
Ch.	Chal.	*Schizotherium*	1	1	1	1	1		Matthew and Granger (1923)
Hsandagolian									
Tapir.	Hela.	*Colodon*			4	4	1	1	Borissiak (1918)
Rhino.	Hyraco.	*Ardynia*				3.4	3	1	Beliayeva (1952)
		*Triplopus?*				?		1	Beliayeva (1954), Radinsky (1967)
		*Allacerops*	4	3	3	3.3		1	Borissiak (1915)
	Para.	*Paraceratherium*	4	3	3	3.3	3	1	Qiu and Wang (2007), Wang, Chang, Meng, and Chen (1981)
		*Dzungariotherium*	4					1	Qiu and Wang (2007), Wang et al. (1981)
		*Turpanotherium* sp.				?		2	
	Amy.	*Cadurcodon*				1.7	1	1	Huang (1982), Wang et al. (1981)
	Rh.	*Aceratherium* sp.				4	2	?	Huang (1982)
		*Aprotodon*				4		1	Wang et al. (1981)
Ch.	Chal.	*Schizotherium*				1	1	1	Borissiak (1920), Gabunia (1951)
Tanbenbulukian									
Rhino.	indet.	*Meschotherium*				?		?	Gabunia (1964)
	Hyraco.	*Ardynia*				3.4	2	2	Qiu, Wang, and Deng (2004)
		*Allacerops*				3.3		1	
	Para.	*Dzungariotherium*	5	4	4	4.3	5	1	Qiu et al. (2004), Qiu (1973), Qiu and Wang (2007), Xu and Wang (1978)
		*Paraceratherium*				3.3		1	Qiu and Wang (2007), Xu and Wang (1978)
		*Turpanotherium*				?		2	Qiu and Wang (2007)
		*Aralotherium*	4	3	3	3.3		1	Gromova (1959), Qiu and Wang (2007), Ye, Meng, and Wu (2003)
		*Benaratherium*				?		1	Gabunia (1964)
	Amy.	*Cadurcotherium*	1			1	1	1	De Bonis (1995), Pilgrim (1910), Pilgrim (1912)
	Rh.	*Aceratherium*	4	4	4	4	2	2	Borissiak (1944)
		*Aprotodon*	4	4	4	4		1	Qiu and Xie (1997), Qiu et al. (2004)
Ch.	Chal.	*Schizotherium*				1	1	1	Qiu et al. (2004), Zhai (1978a)
Xiejian + Shanwangian									
Tapir.	Tap.	*Plesiotapirus*	5	5	5	5	1	1	Qiu, Yan, and Sun (1991)
Rhino.	Para.	*Turpanotherium*			4	4	1	2	Qiu and Wang (2007)
	Rh‐Ace.	*Plesiaceratherium*	4	4	4	4	6	1	Lu, Zheng, Sullivan, and Tan (2016), Young (1937)
		*Aceratherium*				5		?	Pilgrim (1912)
		*Subchilotherium*	5	5	5	5		1	Colbert (1935), Deng and Gao (2006)
		*Brachypotherium*				5		1	Khan, Akhtar, Khan, and Shaheen (2012), Pilgrim (1910)
		*Aprotodon*				4		1	
		*Lartetotherium*				4		1	Chen and Wu (1976), Ginsburg (1974), Qiu et al. (2013)
	Rh‐Rhi.	*"Dicerorhinus"*	5	5	4	4.7	2	1	Pilgrim (1910)
		*Bugtirhinus*	4		4	4		1	Antoine and Welcomme (2000)
	Rh‐Dic.	*Protaceratherium*				5	1	2	Antunes and Ginsburg (1983)
Equ.		*Anchitherium*				5	1	1	Colbert (1939)
Ch.	Chal.	*Phyllotillon*				3	4	1	Forster‐Cooper (1920), Pilgrim (1910), Qiu, Wang, and Xie (1998)
		*Chalicotherium*				1		1	Forster‐Cooper (1920)
		*Anisodon*				1		1	
		*Borissiakia*		1	1	1		1	Borissiak (1946), Butler (1965)
Tunggurian									
Tapir.	Tap.	*Plesiotapirus*				5	1	1	Qiu et al. (1991)
Rhino.	Rh‐Ace.	*Aceratherium*				5	5	2	
		*Subchilotherium*				5		1	Colbert (1935), Deng and Gao (2006)
		*Brachypotherium*				5		1	
		*Plesiaceratherium*				4		1	
		*Acerorhinus*	5	5	5	5		1	Cerdeño (1996)
	Rh‐Rhi.	*Hispanotherium*	4	4	4	4	7	3	Cerdeño (1996), Zhai (1978b)
		*H*. (*"Tesselodon"*)						3	Yan (1979)
		*H*.(*"Caementodon"*)						3	Guan (1988)
		*H*.(*"Huaqingtherium"*)						？	Guan (1993)
		*H*. (*"Beliajevina"*)						3	Heissig (1974)
		*Shennongtherium*	4	4	4	4		？	Huang and Yan (1983)
		*Alicornops*	5	5	5	5		1	Deng (2006a)
Equ.		*Anchitherium*				5	1	1	
Ch.	Chal.	*Anisodon*				1	2	1	
		*Chalicotherium*	1	1	1	1		1	Colbert (1934)
Bahean									
Tapir.	Tap.	*Tapirus*	5	5	5	5	1	1	Deng, He, and Chen (2008)
Rhino.	Rh‐Ace.	*Chilotherium*				?	4	2	Deng (2006b)
		*Subchilotherium*				5		1	Deng and Gao (2006)
		*Acerorhinus*				5		1	
		*Brachypotherium*				5		1	
	Rh‐Rhi.	*Gaindatherium*	5	5	5	5	6	1	Colbert and Brown (1934)
		*Parelasmotherium*	4	4	4	4		3	Deng (2001b), Deng (2007)
		*Sinotherium*	4	4	4	4		3	Ringström (1923)
		*Ningxiatherium*	4	4	4	4		3	Chen (1977), Deng (2008)
		*Diceros*	5	5	5	5		1	Deng and Qiu (2007)
		*Dicerorhinus*				5		1	Savage and Russell (1983)
Equ.		*Anchitherium*				5	5	1	
		*Sinohippus*	5	5	5	5		1	Hou, Deng, He, and Chen (2007), Zhai (1962)
		*Cormohipparion*				5		3	MacFadden and Bakr (1979)
		*Hipparion* (*Hipparion*)				5		3	Qiu and Xie (1998), Qiu, Huang, and Guo (1987)
		*H*. (*Sivalhippus*)				5		3	Sun et al. (2018a)
Ch.	Chal.	*Chalicotherium*	1	1	1	1	4	1	Colbert (1934)
		*Nestoritherium*	1	1	1	1		1	Chen, Deng, He, and Chen (2012)
		*Ancylotherium*				1		1	
		*Anisodon*				1		1	
Baodean									
Tapir.	Tap.	*Tapirus*	5	5	5	5	1	1	Ji et al. (2015)
Rhino.	Rh‐Ace.	*Chilotherium*	4	4	4	4	4	2	Deng (2001a), Sun, Li, and Deng (2018b)
		*Dihoplus*				4		2	Pandolfi, Gasparik, and Piras (2015)
		*Shansirhinus*	5	4	4	4.3		2	Deng (2005a)
		*Acerorhinus*				5		1	
	Rh‐Rhi.	*Iranotherium*	4	4	4	4	4	3	Deng (2005b)
		*Sinotherium*				4		3	
		*Dicerorhinus*				5		1	Deng and Chen (2016), Ringström (1924)
		*Rhinoceros*				5		1	Colbert (1935), Falconer and Cautley (1847), Zin Maung Maung et al. (2010)
Equ.		*Sinohippus*				5	7	1	
		*Hipparion* (*Hipparion*)				5		3	Qiu et al. (1987)
		*H*. (*Sivalhippus*)				5		3	Sun et al. (2018a)
		*H*. (*Hippotherium*)						3	Sun (2018)
		*H*. (*Cremohipparion*)						3	Sun (2018)
		*H*. (*Baryhipparion*)						3	
		*Shanxihippus*				5		3	Bernor et al. (2018)
Ch.	Chal.	*Nestoritherium*				1	2	1	Xue and Coombs (1985)
		*Ancylotherium*				1		1	
		*A*. (*="Huanghotherium"*)				?			Tong, Huang, and Qiu (1975)
		*A*. (*="Gansodon"*)							Wu and Chen (1976)
Gaozhuangian									
Tapir.	Tap.	*Tapirus*				5	1	1	
Rhino.	Rh‐Ace.	*Dihoplus*				4	2	2	
		*Shansirhinus*				4.3		2	
Equ.		*Hipparion* (*Hipparion*)				5	5	3	
		*H*. (*Plesiohipparion*)						3	
		*H*. (*Cremohipparion*)						3	
		*H*. (*Baryhipparion*)						3	
		*Proboscidipparioon*				5		3	Deng (2012)
Ch.	Chal.	*Ancylotherium*				1	1	1	
Mazegouan									
Tapir.	Tap.	*Tapirus*				5	1	1	
Rhino.	Rh‐Ace.	*Dihoplus*				4	1	2	
	Rh‐Rhi.	*Dicerorhinus*				5	3	1	Zin Maung Maung et al. (2010)
		*Rhinoceros*				5		1	
		*Coelodonta*	4	4	4	4		3	Deng et al. (2011)
Equ.		*Hipparion* (*Plesiohipparion*)				5	4	3	
		*H*. (*Baryhipparion*)						3	
		*Proboscidipparioon*				5		3	
		*Plesippus*				5		3	Sun (2018)
Quaternary									
Tapir.	Tap.	*Tapirus*				5	2	1	
		*Megatapirus*	5	5	5	5		1	Colbert and Hooijer (1953)
Rhino.	Rh‐Rhi.	*Dicerorhinus*				5	4	1	
		*Rhinoceros*				5		1	Yan, Wang, Jin, and Mead (2014), Zin Maung Maung et al. (2010)
		*Coelodonta*	4	4	5	4.3		3	Deng (2002)
		*Stephanorhinus*				5		2	Tong and Wu (2010)
Equ.		*Hipparion (Plesiohipparion)*				5	4	3	
		*Proboscidipparioon*				5		3	Deng (2012)
		*Equus*				5		3	
		*Plesippus*				5		3	Sun (2018)
Ch.	Chal.	*Hesperotherium*			1	1	2	1	Qiu (2002), Tong (2006)
		*Nestoritherium*	1	1	1	1		1	Falconer (1868)

Note

Almost all Eocene perissodactyls from Asia were considered to have brachydont teeth, which were left blank in the table.

Abbreviations: Ace., Aceratheriinae; Amy., Amynodontidae; Bron., Brontotheriidae; Ch., Chalicotherioidea; Chal., Chalicotheriidae; Dep., Deperetellidae; Dic., Diceratheriinae; Eomo., Eomoropidae; Equ., Equoidea; H., Hypsodonty; Hela., Helaletidae; Isecto., Isectolophidae; Hy., Hyrachyidae; Hyraco., Hyracodontidae; Loph., Lophialetidae; M., mean value of degree of premolar molarization; N., number of genera in each family; Palae., Palaeotheriidae; Para., Paraceratheriidae; Rh., Rhinocerotidae; Rhi., Rhinocerotinae; Rhino., Rhinocerotoidea; Tap., Tapiridae; Tapir., Tapiroidea.

**TABLE A2:** The mean value of premolar molarization degrees in different perissodactyl families through the post‐Paleocene Asian Land Mammal Ages (ALMA)

ALMA	Isecto.	Hela. + Tapir.	Loph.	De.	Hy.	Hyraco.	Para.	Amy.	Rhino.	Bron.	Chali.
Bumbanian	1	1	1			1				1	1
Arshantan	1	1	1	1	3	3.3	3			1	1
Irdinmanhan	1	2.5	1.1	4.5	3.3	1.4	3.3	1		2.1	1
Sharamurunian		2.5	?	4.7	1	1.5	3.1	1.7	4	3	1
Ulangochuian				4		2	2.9	2.3	4	4.4	1
Ergilian		3				2.2	3.5	1.6	3.2	3.6	1
Hsandagolian		4				3.4	3.3	1.7	4		1
Tanbenbulukian						3.4	3.6	1	4		1
Xiejian + Shan‐wangian		5					4		4.5		1.5
Tunggurian		5							4.6		1
Bahean		5							4.7		1
Baodean		5							4.4		1
Gaozhuangian		5							4.2		1
Mazegouan		5							4.5		
Quaternary		5							4.8		1

Abbreviations: Amy., Amynodontidae; Bron., Brontotheriidae; Chali., Chalicotherioidea; De. Deperetellidae; Hela., Helaletidae; Hy., Hyrachyidae; Hyraco., Hyracodontidae; Loph., Lophialetidae; Para., Paraceratheriidae; Rhino., Rhinocerotidae; Tapir., Tapiridae.

**TABLE A3:** Generic numbers of different perissodactyl groups through the post‐Paleocene Asian Land Mammal Ages (ALMA)

ALMA	Tapiroidea	Rhinocerotoidea	Equoidea	Brontotheriidae	Chalicoth‐erioidea
Bumbanian	10	3	2	1	2
Arshantan	6	7	1	4	2
Irdinmanhan	14	14	1	14	2
Sharamurunian	7	19	1	4	2
Ulangochuian	1	8	0	3	1
Ergilian	1	12	1	6	1
Hsandagolian	1	9	0	0	1
Tanbenbulukian	0	10	0	0	1
Xiejian‐Shanwangia	1	10	1	0	4
Tunggurian	1	12	1	0	2
Bahean	1	10	5	0	4
Baodean	1	8	7	0	2
Gaozhuangian	1	2	5	0	1
Mazegouan	1	4	4	0	0
Quaternary	2	4	4	0	2

**TABLE A4:** Generic numbers of three classes of hypsodonty through the post‐Paleocene Asian Land Mammal Ages (ALMA)

ALMA	Brachydont	Mesodont	Hypsodont
Bumbanian	18	0	0
Arshantan	20	0	0
Irdinmanhan	45	0	0
Sharamurunian	32	1	0
Ulangochuian	12	1	0
Ergilian	20	0	1
Hsandagolian	9	1	0
Tanbenbulukian	8	3	0
Xiejian‐Shanwangia	13	2	0
Tunggurian	9	1	4
Bahean	13	1	6
Baodean	7	3	8
Gaozhuangian	2	2	5
Mazegouan	3	1	5
Quaternary	6	1	5
